# New Insights Into Immunological Therapy for Retinal Disorders

**DOI:** 10.3389/fimmu.2020.01431

**Published:** 2020-07-03

**Authors:** Atsunobu Takeda, Ryoji Yanai, Yusuke Murakami, Mitsuru Arima, Koh-Hei Sonoda

**Affiliations:** ^1^Department of Ophthalmology, Graduate School of Medical Sciences, Kyushu University, Fukuoka, Japan; ^2^Department of Ophthalmology, Clinical Research Institute, Kyushu Medical Center, National Hospital Organization, Fukuoka, Japan; ^3^Department of Ophthalmology, Graduate School of Medicine, Yamaguchi University, Yamaguchi, Japan

**Keywords:** immune privilege, non-infectious uveitis, diabetic retinopathy, retinopathy of prematurity, retinitis pigmentosa, vitreoretinal lymphoma

## Abstract

In the twentieth century, a conspicuous lack of effective treatment strategies existed for managing several retinal disorders, including age-related macular degeneration; diabetic retinopathy (DR); retinopathy of prematurity (ROP); retinitis pigmentosa (RP); uveitis, including Behçet's disease; and vitreoretinal lymphoma (VRL). However, in the first decade of this century, advances in biomedicine have provided new treatment strategies in the field of ophthalmology, particularly biologics that target vascular endothelial growth factor or tumor necrosis factor (TNF)-α. Furthermore, clinical trials on gene therapy specifically for patients with autosomal recessive or X-linked RP have commenced. The overall survival rates of patients with VRL have improved, owing to earlier diagnoses and better treatment strategies. However, some unresolved problems remain such as primary or secondary non-response to biologics or chemotherapy, and the lack of adequate strategies for treating most RP patients. In this review, we provide an overview of the immunological mechanisms of the eye under normal conditions and in several retinal disorders, including uveitis, DR, ROP, RP, and VRL. In addition, we discuss recent studies that describe the inflammatory responses that occur during the course of these retinal disorders to provide new insights into their diagnosis and treatment.

## Introduction

In the last 2 decades, advances in the interdisciplinary collaboration of the fields of molecular biology, biochemistry, genetics, and biomedicine have resulted in tremendous breakthroughs in the treatment of refractory ocular disorders. Infliximab (IFX), a chimeric antibody of the tumor necrosis factor (TNF)-α, is a biologics that is used for treating ocular symptoms of Behçet's disease that have not been adequately controlled (1). Anti-vascular endothelial factor (VEGF) agents such as ranibizumab and aflibercept are used as the first-line therapy in the management of intractable retinal disorders such as neovascular age-related macular degeneration and diabetic macular edema (DME). These agents can also maintain remission in such cases (2, 3). For a long time, laser photocoagulation alone has been used for the primary treatment of etinopathy of prematurity (ROP). However, in 2018, ranibizumab was also validated for the treatment of ROP in Japan. Gene therapy clinical trials targeting the treatment of autosomal recessive or X-linked retinitis pigmentosa (RP), which is an incurable genetic retinal disorder, have been initiated (4). Furthermore, the overall survival of patients with vitreoretinal lymphoma (VRL), which is a fetal retinal malignancy, has increased because of improved treatment strategies that involve intense systemic chemotherapy and/or radiotherapy (5–7). However, unmet needs remain in the management of these retinal disorders because of primary and secondary treatment failure or non-response to the biologics, or most cases of RP untargeted by gene therapy.

The eye, just like the brain and the testes, is an immune-privilege site (8). Ocular immune privilege is an active process in which the regulatory molecules and cells of the eye modulate the induction and the expression of inflammation (8–10). As long as the ocular immune-privilege system is working, a harmful immune response and degenerative eye diseases can be prevented. By limiting intraocular inflammation, immune privilege preserves the integrity of the visual axis and thereby prevents blindness (11). However, the mechanism of immune privilege can be compromised genetically and/or by environmental stimuli such as damage-associated molecular patterns, infection, and a chronic immune response, and thereby give rise to various retinal disorders (11).

Immunological responses to various environmental stimuli have been associated with the pathogenesis of uveitis and retinal vascular diseases such as diabetic retinopathy (DR) and ROP (11–13). Retinitis pigmentosa is a genetic disorder, although inflammatory responses to microenvironmental changes, such as rod cell death, which occur after the primary onset of the disease, may cause subsequent loss of cone cells (11). In VRL, oncogenic mutations of VRL cells and the evasion of immune surveillance because of the immunosuppressive ocular microenvironment may contribute to tumor growth.

In this review, we focus on the roles of immunological responses in a normal conditions and in several major retinal disorders including non-infectious uveitis (NIU), DR, ROP, RP, and VRL. In addition, we contemplate new approaches for the diagnosis and treatment of these intractable retinal disorders from an immunological point of view.

## The Normal Immunological Condition in the Eye

A properly elicited and regulated immune response by the human body is necessary for eliminating threats due to infectious microbes and tissue trauma to avoid irreversible tissue damage (11). Acute inflammation should be self-limiting and normally attenuated after the elimination of deleterious stimuli to enable physiological recovery. Chronic inflammation causes degenerative diseases with consequent loss of organ function.

Ocular immune privilege is believed to elicit self-limiting immune responses (14). Several soluble and cell-bound inhibitory factors are involved in the mechanism of ocular immune privilege to create an intraocular immunosuppressive microenvironment, which prevents excessive immune activation and subsequent tissue damage. These factors include transforming growth factor-beta (TGF-β)2 (15), retinoic acid (16), and multiple immunosuppressive factors in ocular fluids (17), and the constitutive expression of the Fas ligand (18), programmed death-ligand 1 (PD-L1) (19), galectins (20), membrane glycoprotein CD200 receptor 1 (21), cytotoxic T lymphocyte-associated protein (CTLA)-2a (22), B7 (23, 24) on the surface of ocular cells. For the maintenance of retinal homeostasis, these immunosuppressive molecules in the eye actively regulate the induction and the expression of inflammation to prevent excessive activation and subsequent tissue damage.

In addition to the abovementioned local immunosuppression, ocular immune privilege is associated with the development of a type of an antigen-specific systemic immune regulation, called “anterior chamber-associated immune deviation” (“ACAID”) (8, 25). ACAID is induced by intrinsic intraocular bone marrow-derived antigen-presenting cells (APCs) that trap an antigen within the anterior chamber and migrates to the spleen via circulating blood (8, 26). Antigens from the anterior chamber can be transported to the regional lymph nodes (27). Tolerogenic CD11c^+^ dendritic cells (DCs) also transport antigens to the thymus (27). APCs bearing the eye-derived antigen can elicit the development regulatory T (Treg) cells in these lymphoid organs. In the eye, innate immune cells such as microglial cells, neutrophils, monocyte-macrophages, natural killer (NK) cells, natural killer T (NKT) cells, and γδT cells can exhibit a broad range of antigen recognition and confer a widely distributed form of immunity, which constitutes the first line of defense against various invading pathogens (28).

The anterior chamber and the vitreous cavity or the posterior chamber of the eye have the capacity to induce systemic immune deviation (29). This phenomenon is called “vitreous cavity-associated systemic immune deviation” (“VCAID”). Research demonstrates that F4/80^+^ hyalocytes are distributed over the retinal surface (30) and that murine hyalocytes are bone marrow-derived and turned over in 4 months (31). Hyalocytes have strong scavenger activity (30); therefore, we postulated that ocular hyalocytes capture an antigen on the retinal surface and carry it via the blood to the spleen, which induces antigen-specific Tregs (30).

## Non-Infectious Uveitis (NIU)

### Human Non-infectious Uveitis

Non-infectious uveitis (NIU) is a sight-threatening disorder associated with systemic autoimmune diseases such as Behcet's disease, sarcoidosis, and Vogt–Koyanagi–Harada disease (32). NIU is often recurrent and causes tissue destruction and scarring, especially in the retina and uvea, which results in permanent loss of vision. Early studies showed that T-helper (Th) 1 and Th17 cells are the major effector cells and are crucial for development of uveitis (33). In many cases, the administration of several immunosuppressive drugs for an extended period is necessary to control inflammation in eyes with uveitis (34). These agents include corticosteroids, tacrolimus, and cyclosporine, which have a strong T cell-suppressive effect and have serious side effects, such as diabetes, hypertension, and nephrotoxicity (35, 36). A new generation of biological compounds that inhibit T cell activation such as monoclonal antibodies and recombinant forms of natural inhibitory molecules have emerged (36, 37). For example, IFX is validated for the treatment of refractory ocular Behçet's disease (1, 38, 39). Adalimumab (ADA), which is a recombinant human immunoglobulin G1 monoclonal antibody to TNF-α, has also been validated for the treatment of patients with NIU in the United States and in Japan. ADA has been proven as an effective corticosteroid-sparing agent that reduces adverse effects associated with long-term usage of corticosteroids (34). Immunosuppressive therapeutic agents for treating non-infections uveitis (based on the guidelines of an international expert steering committee consisting of uveitis specialists are summarized in Table 1) (40). However, several studies report that IFX can cause the development of autoimmune diseases, primarily cutaneous vasculitis, lupus-like syndrome, and malignant lymphoma (41). In recent reports, some patients had a decreased response to IFX or ADA during the course of the treatment, owing to the development of antidrug antibodies against IFX or ADA (42–44). Therefore, new effective therapeutic targets for uveitis with less severe adverse effects need to be identified.

**Table 1:** Systemic corticosteroid and immunomodulatory therapeutic agents for non-infectious uveitis.

**Drugs**	**Drug administration route and dosage**	**Disease entities or cause**	**Evidence level (No. of publications)**
Corticosteroid	Orally prednisolone, 20–60 mg/day Intravenously methylprednisolone, 1,000 mg/day Tapering to low-dose oral prednisone and addition of a corticosteroid sparing agent	NIU	
Mycophenolate preparations	Oral, 500–3,000 mg/day	NIU BCR VKH disease	2B^¶^ 2B/3 2B/3
Azathioprine^§^	Started at 1 mg/kg/day and increased to 2–3 mg/kg/day in steps of 50 mg every 2 weeks	NIU BD VKH disease	2B 2B 4
Methotrexate	Adult: oral, 6–25 mg/week Child: oral, 4–10 mg/week	NIU VKH disease	2B 2B/3
Cyclophosphamide	Oral, 20–100 mg/day Intravenos, 750–1,000 mg/m^2^ of body surface area monthly infusions	NIU	4
Tacrolimus	Oral, 0.12–0.3 mg/kg	NIU	2B
Cyclosporine	Oral, 3–5 mg/kg	NIU	2B
Infliximab	Intravenous, 5 mg/kg at weeks 0, 2, and 6, and every 8 weeks thereafter	BD, BCR, sarcoidosis, idiopathic vasculitis, VKH disease Pediatric NIU (uveitis entities include JIA, BD, sarcoidosis, VKH disease)	2B (2), 3B (1), 4 (4) 2B (1), 4 (2), 5 (1)
Adalimumab	Initial dose of 80 mg, followed by 40 mg administered every other week starting 1 week after the initial dose	NIU (including different uveitis entities: BD, idiopathic uveitis, sarcoidosis, BRC, TINU, VKH disease, pars planitis; other: HLA-B27, JIA)	1B (4), 2B (4), 4 (5), 5 (2)

*NIU, non-infectious uveitis; BCR, birdshot chorioretinopathy; VKH, Vogt–Koyanagi–Harada disease; BD, Behçet's disease; JIA, juvenile idiopathic arthritis; TINU, tubulointerstitial nephritis and uveitis*.

¶ *Evidence level 4 and grade C recommendation for mycophenolate sodium*.

§ *Includes one study with methotrexate and mycophenolate mofetil as comparators*.

### Experimental Autoimmune Uveitis

Experimental autoimmune uveitis (EAU) is an animal disease model of a T cell–mediated autoimmune disease that mimics many of the pathological features of human uveitis (45, 46). EAU is induced by injecting animals with purified retinal antigens such as S-Ag, a fragment (residues 1–20, GPTHLFQPSLVLDMAKVLLD) of the human interphotoreceptor retinoid-binding protein (hIRBP); rhodopsin (or opsin); phosducin, or recoverin. The disease is mediated by Th1 and Th17 cells (47, 48), which are generated from naive T cells in response to their exposure to proinflammatory cytokines and to the foreign antigens presented by APCs—including DCs, macrophages, NK cells, and B lymphocytes—in secondary lymphoid organs. Infiltration of inflammatory cells such as granulocytes and macrophages and other non-specific lymphocytes that can destroy ocular tissue also contributes to the development of EAU (48, 49). In the next section, we will discuss recent studies of EAU model mice and review studies on microglia, NK cells, NKT cells, DCs, the P2X7 receptor, Notch signaling, and the transcription factor Foxp3 (forkhead box P3), which have been identified as targets for translational medicine. Such research has provided insight into the mechanisms underlying disease pathogenesis and the basis for the development of new preventive or therapeutic approaches to human uveitis.

### Advances in the Treatment of EAU

The important role of microglia in the regulation of inflammatory cell infiltration into the retina associated with the initiation of retinal autoimmune uveitis has recently been demonstrated (50). During the early phase of EAU (days 7–10), microglia have a direct effect on the increase in the number of various adherent vascular leukocytes, including T cells, major histocompatibility complex (MHC) class II^+^ cells, and CD11b^+^ cells. This effect was specific to microglia, given that it was not reproduced by other immune cell types such as monocytes-macrophages. Therefore, immunomodulatory therapies targeted at microglia are a primary focus in the development of new treatments for patients with uveitis.

We previously explained that NKT cells suppress the induction of Th17 cells and the ocular infiltration of hIRBP-specific T cells in EAU. However, in contrast to the ameliorating effects of NKT cell activation that is apparent during the initiation phase of EAU, the activation during the effector phase exacerbates disease pathology. This finding suggests that NKT cells have a dual role in EAU, depending on the phase of the disease (51).

CD83^+^CCR7^+^ NK cells induced by interleukin (IL)-18 released from DCs promote EAU (52). NK cells are negatively regulated by a soluble form of CD83 in EAU (53). In addition, DC–NK cell interactions that underlie the regulation of Th1 responses modulate the adaptive Th17 response and limit tissue-specific autoimmunity through the innate interferon (IFN)-γ-IL-27 axis in this model (54).

Treg cells are necessary for the resolution of EAU and the prevention of relapse (55, 56). A recent study demonstrated that PD-1^+^CD25^+^CD4^+^ Treg cells require programmed cell death 1 (PD-1) stimulation through a melanocortin-adenosine pathway to suppress EAU. These Treg cells did not induce suppressor activity in APCs through the PD-1 pathway (57).

Many studies have demonstrated that monocytes-macrophages have a role in the development of EAU (58). Activated bone marrow–derived macrophages are required during the effector phase of EAU (45). However, in addition to their proinflammatory function, macrophages have suppressive effects on ocular inflammation, especially in the chronic phase. Suppressor of cytokine signaling 3 (SOCS3) in macrophages was recently found to be important in the suppressing the inflammation caused by these cells in EAU (59).

Extracellular adenosine triphosphate (ATP) is a key chemotactic signal for the recruitment of innate immune cells to sites of brain injury (60). ATP is actively released via exocytosis or transporters during the early phase of apoptotic cell death, whereas it is passively released from necrotic cells after the rupture of the plasma membrane. Extracellular ATP acts at P2X and P2Y purinergic receptors and induces the formation of inflammasomes, which are large intracellular multiprotein complexes that are key players in host defense during the innate immune response (61). The P2X receptor family consists of ligand-gated cation channels that open in response to the binding of extracellular ATP. Among the seven mammalian P2X receptors, P2RX7 shows the highest affinity for ATP and is highly expressed in immune cells such as monocytes and T lymphocytes (62, 63). Research has demonstrated that the genetic ablation of P2RX7 or the administration of the P2RX7 antagonist BBG in mice suppresses EAU clinically and histopathologically by attenuating hIRBP-dependent induction of interferon (IFN)-γ and IL-17 (64).

### Dendritic Cells in the Eye

DCs are highly efficient APCs and have the unique ability to prime and activate naive T lymphocytes (65, 66). They are divided into three types, based on their function: immature DCs, mature DCs, and regulatory DCs. Under physiological conditions, DCs are widely distributed among tissues and organs (67) where they are in an immature state and contribute to immune surveillance. In the eyes of mice and rats, these cells are at the peripheral margin and in juxtapapillary areas of the retina (68, 69). The functions of DCs in the quiescent retina include promoting the generation of Foxp3^+^ Treg cells and inhibiting the activation of naive T cells induced by splenic DCs and antigens (69).

### Dendritic Cells in EAU

DCs have an essential role in innate immunity. They also link the innate and adaptive immune systems and are key for the induction of late immune responses. Cell-based therapy involving the *ex vivo* manipulation of mature or regulatory DCs has been adopted as a means to induce tolerance in autoimmune disease (70–72). Studies of the mechanisms of DC function in uveitis are thus warranted to identify new therapeutic targets for this condition. Mature DCs pulsed with uveitogenic antigens induce the development of EAU (69). Treatment with fixed immature DCs, but not with fixed mature DCs, has also been demonstrated to ameliorate the progression of EAU by inhibiting uveitogenic CD4^+^ T cell activation and differentiation (73). In addition, impairment DC maturation with drugs prevents the generation of antigen-specific Th1 and Th17 cells and thereby attenuates EAU (74). Moreover, regulatory DCs induced *in vitro* suppress the development of EAU (75). These various data altogether indicate that the regulation of DC status is potentially beneficial for the treatment of uveitis.

**Figure 1:**
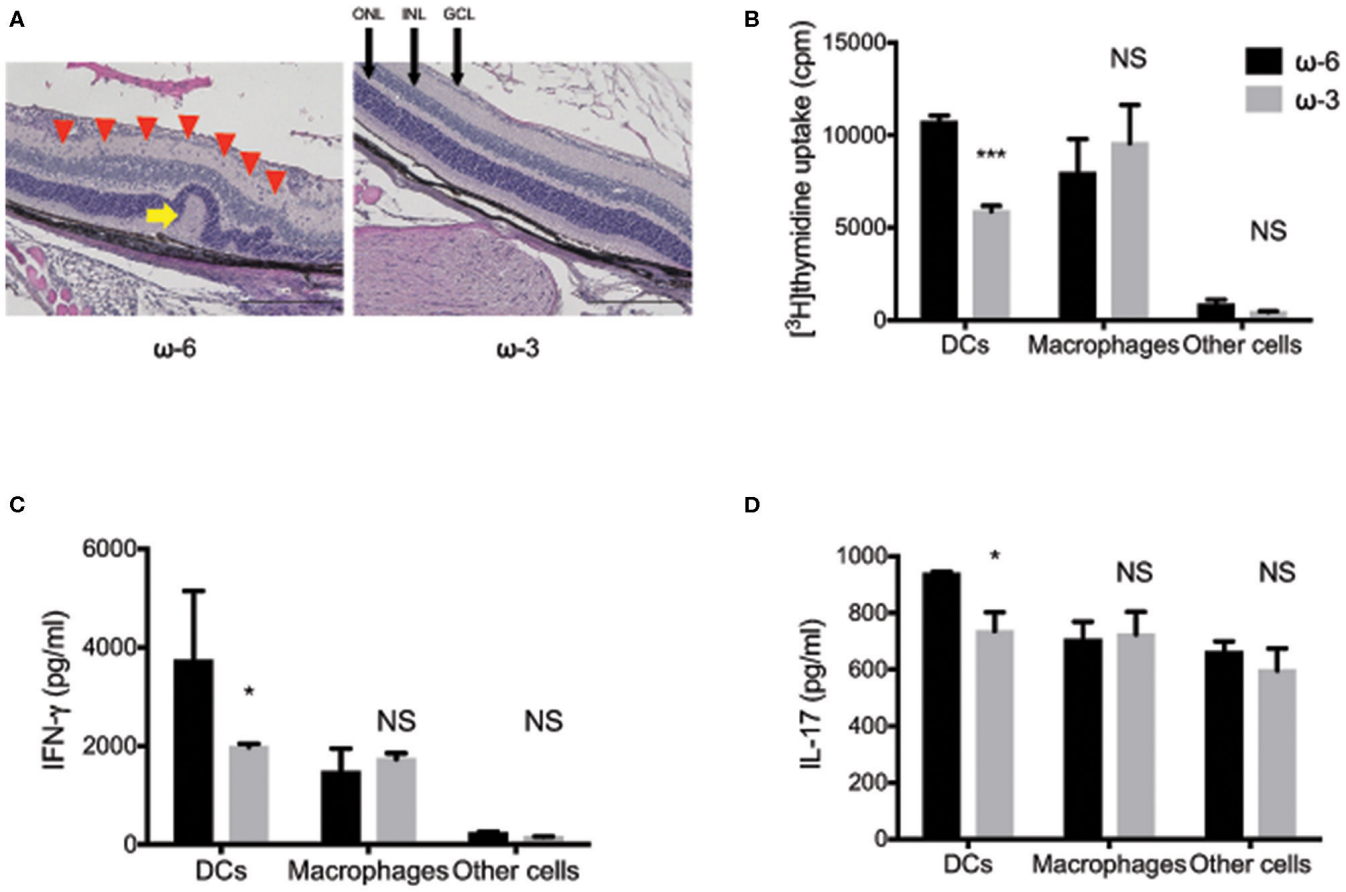
The effects of ω-3 long chain polyunsaturated fatty acids in experimental autoimmune uveitis model mice. **(A)** Hematoxylin-eosin staining of retinal sections at 17 days after disease induction in experimental autoimmune uveitis (EAU) mice maintained on a diet enriched with ω-3 or ω-6 long-chain polyunsaturated fatty acids (LCPUFAs). A red arrowhead indicate inflammatory cells in the retina. A yellow arrow indicates a retinal fold. GCL, ganglion cell layer; INL, inner nuclear layer; ONL, outer nuclear layer. Scale bars, 200 μm. **(B–D)** The proliferation of T cells, as assessed by the measurement of [^3^H]thymidine incorporation **(B)**, and by the production of interferon-γ **(C)**, and interleukin-17 **(D)** in co-cultures of CD4^+^ T cells from EAU mice and the indicated antigen presenting cell fractions from mice fed with ω-3 or ω-6 LCPUFAs. Data are expressed as the means + the standard error of the mean (SEM). ^*^P < 0.05, ^***^P < 0.001; NS, not significant vs. the corresponding value for the ω-6 LCPUFA diet (i.e., Sidak's multiple comparison test). The figure is reproduced from (77) with permission.

In a previous study, conducted by the authors of the present review, we found that mouse spleen-derived DCs mediate the anti-inflammatory action of dietary ω-3 long-chain polyunsaturated fatty acids (LCPUFAs) in EAU (76). Histological analysis at 17 days after disease induction revealed retinal folds and immune cell infiltration in the eyes of EAU mice that received DCs from ω-6 LCPUFA–fed mice, and showed that such changes were markedly suppressed in EAU mice that received DCs from ω-3 LCPUFA–fed mice (Figure 1A) (77). Furthermore, DCs exposed to ω-3 LCPUFAs *in vivo* or *in vitro* suppressed T cell proliferation. This finding suggested that ω-3 LCPUFA–treated DCs attenuate inflammation mediated by T cells (Figure 1B). Cytokines released by activated DCs are essential for T cell differentiation, with IL-12 p70 promoting Th1 cell differentiation and with IL-6 and TGF–β promoting Th17 cell differentiation (78, 79). We also found that dietary ω-3 LCPUFAs acting via adoptively transferred DCs markedly inhibited IL-12 p70 and IL-6 production by T cells from EAU mice. This finding is consistent with the notion that ω-3 LCPUFAs suppress Th1 and Th17 cytokine production by CD4^+^ T cells, through the mediation of DCs (Figures 1C,D). Moreover, we also found that ω-3 LCPUFAs, acting via DCs, suppressed the production of proinflammatory cytokines and the anti-inflammatory cytokine IL-10. However, the DC-dependent anti-inflammatory effects of ω-3 LCPUFAs appear to outweigh their proinflammatory effects, at least in EAU.

## Diabetic Retinopathy

The global prevalence of diabetes mellitus (DM) tends to increase yearly, and the number of DM patients is estimated to reach 592 million within 20 years (80). Diabetic retinopathy (DR) is a representative microvascular complication of DM that causes visual impairment in working-age adults (81). Leasher et al. reported that blindness and moderate to severe visual impairment due to DR increased in 20 years (1990–2010) from 2.1 to 2.6% and from 1.3 to 1.9%, respectively (82). Vascular abnormalities such as hemorrhage, microaneurysm, capillary non-perfusion, and exudates are frequently observed in DR. Therefore, DR has been perceived as a disease that originates from vascular abnormalities. However, several lines of evidence indicate an association between inflammation and the pathophysiology of DR (12). The principal causes of visual impairment in DR are proliferative DR (PDR) and DME. In the next section, we will discuss the relationship between inflammation and PDR or DME formation.

### Inflammation in PDR

PDR is characterized by the development of preretinal neovascularization and epiretinal fibrovascular membranes (FVMs) (81). Prolonged hyperglycemia, accumulation of advanced glycation end products, and oxidative stress under diabetic condition induce VEGF expression in the retina through protein kinase C activation. VEGF promotes leukostasis by increasing the expression of intercellular adhesion molecule-1 (ICAM-1) in retinal vascular endothelial cells (83). Low-grade inflammation initiated by leukocytes (e.g., monocytes and granulocytes) that adhere to endothelial cells via ICAM-1 induces vascular endothelial cell damage and cell death and promotes capillary loss and infiltration of leukocytes into the retina (84). Expanding inflammation and ischemia induces the further expression of VEGF and other angiogenic cytokines such as TNFα, IL-1β, IL-6, and IL-8. This negative cycle gradually advances DR (85). Van Hecke et al. showed that the prevalence of DR is positively associated with the serum levels of C-reactive protein (CRP) and soluble ICAM-1 (sICAM-1), which suggests that early vascular damage is actually caused by inflammation (86). Preventing the onset of DR is important to avoid DR-induced visual impairment. Therefore, the serum levels of CRP or sICAM-1 that indicate the level of the inflammatory activity may become useful biomarkers for predicting the onset of DR.

**Figure 2:**
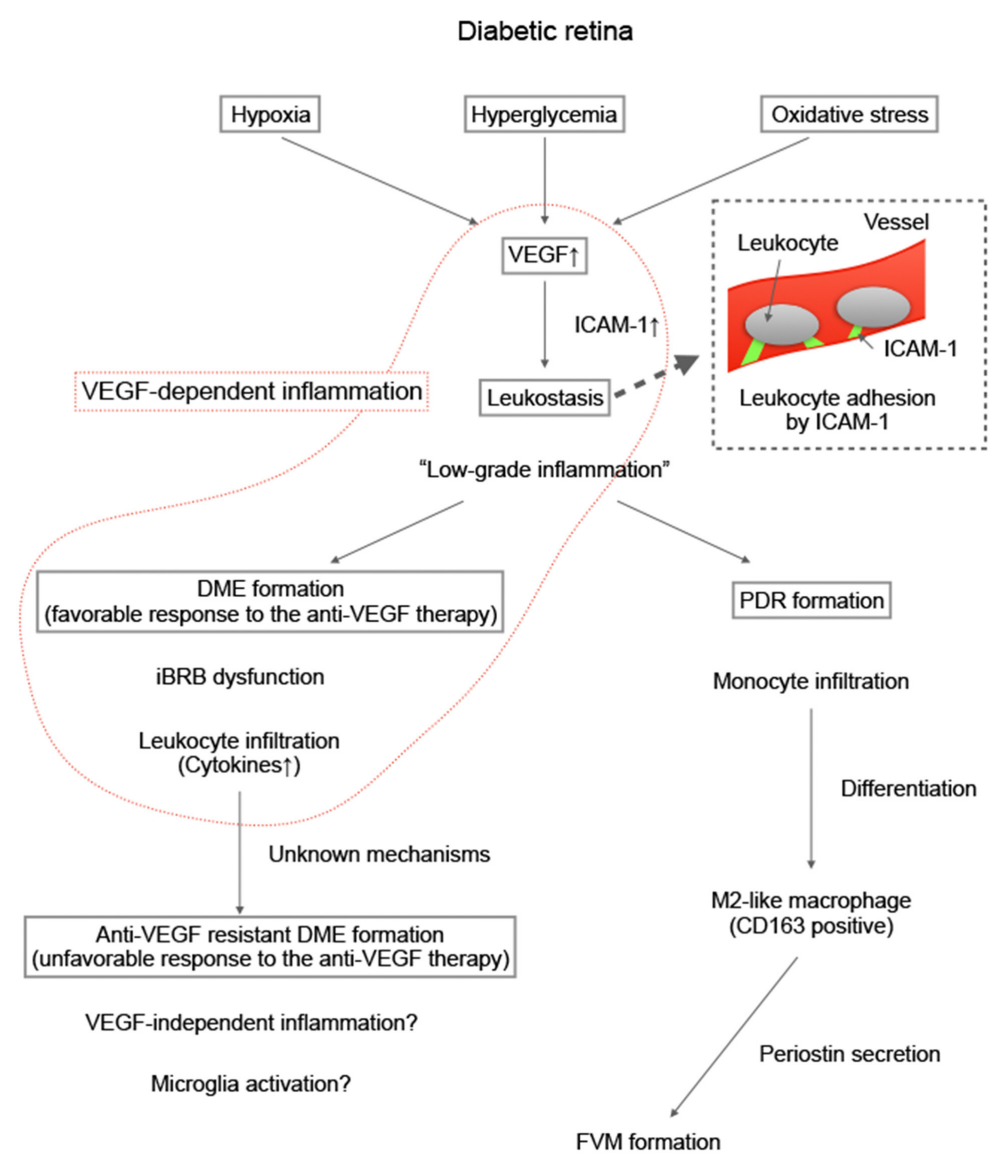
Summary of our hypothesis on the formation of proliferative diabetic retinopathy and antivascular endothelial growth factor resistant diabetic macular edema. Diabetic macular edema (DME) animal models such as Akimba mouse models have been developed. The details of the involvement of inflammation in the pathogenesis of DME are expected to be identified in the future.

In a previous study with an experimental animal model, we previously reported that intravitreal injection of an anti-VEGF agent can attenuate the infiltration of leukocytes, especially macrophages, into the retina and can suppress preretinal neovascularization (87). Esser et al. demonstrated that inflammatory phase macrophages are localized in FVMs in PDR (88). This finding implied that macrophage-induced inflammation is associated with FVM formation. However, macrophages have diverse populations, and the role of each population differs (89). With regard to various populations of macrophages, Zhou et al. found that M2-like macrophages (i.e., CD163-positive macrophages) promote pre-retinal angiogenesis in DR (90). Kobayashi et al. also reported that some M2-like macrophages localized in the FVMs produce periostin (91). Periostin is a matricellular protein that is essential for FVM formation in PDR (92). Thus, we believe that M2-like macrophages may potentially become a new therapeutic target in treatment of PDR. The process from the onset of DR to the occurrence of PDR is summarized in Figure 2.

### Inflammation in DME

Blood vessels in the central nervous system (CNS), including the retina have a vascular barrier function and maintain a proper neural microenvironment through strict control of vascular permeability (93). Retinal neural tissue is separated from the blood stream by the inner blood-retinal barrier (94). The collapse of the inner blood-retinal barrier under diabetic conditions results in DME, and persistent DME causes irreversible neural damage (94). Experimental investigations have proven that low-grade inflammation after leukostasis can disrupt the vascular barrier (83), and VEGF and some cytokines/chemokines that can increase vascular permeability are secreted from infiltrated leukocytes (94). Moreover, several clinical studies have demonstrated the upregulation of inflammatory cytokines/chemokines in the vitreous fluid of eyes with DME, which suggests a relationship between inflammation and the pathogenesis of DME (95).

VEGF is the most studied molecule that can increase vascular permeability (96). Regular intravitreal injections of anti-VEGF agents (i.e., anti-VEGF therapy) can improve vision and reduce the accumulation of macular fluid in DME. This therapy is the primary treatment modality for DME (3). However, clinical trials have demonstrated that ~40% of patients are anti-VEGF resistant (97). Several studies were conducted to detect additional therapeutic targets for the treatment of DME. Sfikakis et al. demonstrated the therapeutic efficacy of the intravenous injection of IFX, an anti-TNFα antibody (98). Anti-inflammatory treatment for DME produces worthwhile results; however some concerns exist regarding the adverse effects of the therapy because of the need to administer high concentrations of IFX (5 mg/kg) multiple times to patients (98). Gale et al. conducted a clinical trial to evaluate the efficacy of an oral chemokine receptor (CCR) type 2 and type 5 (CCR2/CCR5) dual antagonist for treating DME because CCR2 and CCR5 signaling pathways are associated with vascular leakage, monocyte/macrophage infiltration, and increased VEGF expression in the retina of experimental DR models (99). However, its therapeutic efficacy is inferior to that of monthly intravitreal injections of an anti-VEGF agent (99). No molecule beyond VEGF has been found, although the development of a novel treatment for anti-VEGF–resistant DME is urgently needed.

To identify a new target molecule or a novel biomarker for predicting anti-VEGF resistance, many researchers have examined the relationship between the response to anti-VEGF treatment and the concentration of intraocular inflammatory cytokines/chemokines. Hillier et al. showed that an increase in baseline aqueous ICAM-1 is associated with a favorable anatomic response, whereas an increase in baseline aqueous VEGF is associated with an unfavorable anatomic response (100). Shimura et al. concluded that a favorable response was obtained in patients with increased baseline aqueous VEGF, soluble VEGF receptor-1, monocyte chemoattractant protein-1 (MCP-1), ICAM-1, IL-6, and IP-10 (101). Felfeli et al. compared aqueous cytokine concentrations at baseline and 2 months after anti-VEGF therapy; they reported that the aqueous levels of ICAM-1, MCP-1, placenta growth factor, and TGF-β2 decreased significantly in patients with a favorable response (102).

The results of animal experiments have revealed that VEGF induces endothelial ICAM-1 expression in the early stage of DR (83), and that ICAM-1 expression decreases in endothelial cells in the chronic stage of DR (103). Therefore, DME in the “early” stage with mild vascular injury (which does not indicate a short medical history of DR) may respond well to anti-VEGF therapy. Inflammatory cytokines and VEGF decreased in patients with a favorable response to the anti-VEGF therapy, which suggests that VEGF-dependent inflammation may have primarily contributed to DME formation in these patients. The detailed mechanisms of the association between inflammation and anti-VEGF resistance are not completely understood; however, the change in the quality of inflammation may cause treatment resistance. DME animal models such as Akimba mouse models (104), will provide new insights into the involvement of inflammation in the pathogenesis of DME in future research (Figure 2).

## Retinopathy of Prematurity

Retinopathy of prematurity (ROP) is a retinal vasoproliferative disorder that can lead to childhood blindness (105). Blencowe et al. reported that ROP occurs in 184,700 infants annually worldwide. Among these infants, ~20,000 cases progress to a stage that requires treatment (105). The incidence of ROP onset cases has been declining because neonatal management is improving every year (106) and because of a tendency toward a declining birthrate, especially in developed countries. However, a decrease in the number of cases does not necessarily mean a decrease in the number of treatments. Improved neonatal management indicates an increase in the survival rate of preterm infants. The gestational age (GA) and birth weight (BW) of infants enrolled in major large trials are steadily decreasing (107). Low GA and low BW are common risk factors for ROP progression (108). The risk of an increase in the relative proportion of severe ROP cases that require treatment may occur in the future.

Current primary treatments for ROP are retinal photocoagulation and anti-VEGF therapy. Large clinical trials such as the Early Treatment for Retinopathy Of Prematurity study and the Bevacizumab Eliminates the Angiogenic Threat of Retinopahty of Prematurity (BEAT-ROP) study have proven that both methods are effective (109, 110). However, severe ROP is sometimes accompanied by poor mydriasis and vitreous opacity. In such cases, performing laser photocoagulation in infants with ROP is difficult. Anti-VEGF therapy can be considered the first-line therapy for these patients. The BEAT-ROP study revealed that anti-VEGF therapy (i.e., the administration of bevacizumab) is associated with significantly less recurrence than laser photocoagulation in zone I ROP cases (110). To date, bevacizumab is generally used as an off-label drug in some limited facilities. The efficacy of intravitreal injection of ranibizumab (IVR) was recently proven (111). On account that ROP has been approved as one indication for ranibizumab, more infants with ROP are expected to benefit from anti-VEGF treatment in the future.

### Inflammation in ROP

The recurrence rate of ROP after IVR monotherapy is ~30% (111). The provision of optimal treatment at a proper time is essential to prevent the impairment of visual development in infants with ROP; therefore, the identification of a biomarker of ROP progression is very important. VEGF and many other molecules such as insulin-like growth factor-1, hypoxia-inducible factor-1, and reactive oxygen species, may be involved in the onset of ROP (108); however, Sato et al. reported that the vitreous concentrations of inflammatory cytokines/chemokines are increased in patients with ROP (13), which suggests an association between inflammation and ROP pathogenesis. Lyu et al. showed that high levels of aqueous VEGF and macrophage inflammatory protein (MIP)-1β at baseline were associated with the recurrence rate of ROP after IVR therapy (112). In a previous study, we reported that MIP-1β expression significantly increased in the retina of an ROP animal model (113). MIP-1β, also known as chemokine CC motif ligand 4, is a member of the CC chemokine family. Members of the CC chemokine family are characterized by their ability to direct the migration of leukocytes into the inflamed tissues. MIP-1β is upregulated very quickly after hypoxic stimulation in mouse retina (113). Therefore, its expression level may become a very sensitive sensor of retinal ischemia. The administration of a neutralizing antibody against MIP-1β inhibits physiological angiogenesis (113); therefore, we believe that MIP-1β has the potential to be a useful inflammatory biomarker of ROP progression or recurrence rather than a therapeutic target molecule. Moreover, Matsuda et al. reported that mast cell tryptase (MCT) released from mast cells is involved in angiogenesis in ROP (114). They demonstrated that MCT promotes angiogenesis by inducing the production of MCP-1 and other angiogenic factors from endothelial cells (114). On account that the serum MCT level is elevated in infants with ROP, MCT also has the potential to be a useful biomarker of ROP progression.

## Retinitis Pigmentosa

### Gene Mutations and Microenvironment Alterations in Retinitis Pigmentosa

Retinitis pigmentosa refers to a subgroup of inherited retinal degenerations (IRDs) that cause progressive rod-cone degeneration (115). More than 90 causal genes have been identified for typical RP, and these genes are frequently related to the function, structure, and homeostasis of rod photoreceptor cells. Night blindness due to rod dysfunction and death is an early symptom of RP, followed by visual field constriction and loss of central vision due to secondary cone cell death. RP is a major cause of adult blindness in over one million patients globally; no effective treatment substantially delays the disease progression or restores the vision lost to RP (115).

Recent advances in gene therapy have shed light on the treatment of IRDs. Supplementation of the retinal pigment epithelium-specific 65 kDa protein (RPE65) gene in patients with Leber congenital amaurosis due to RPE65 mutations improves their light sensitivity and performance in the multi-luminance mobility test (116). This therapy has been approved in the United States and in Europe. Gene therapy for RP, which targets autosomal recessive or X-liked mutations (e.g., phosphodiesterase 6B, retinaldehyde-binding protein 1, retinitis pigmentosa GTPase regulator, MER proto-oncogene tyrosine kinase) has been assessed in clinical trials (4). However, a significant number of RP patients may not be indicated for gene therapy because of the following reasons: (1) adeno-associated viral vectors cannot accommodate large genes such as the eyes shut homolog, (2) gene correction or editing of autosomal dominant mutation *in vivo* is still challenging, and (3) many patients are first diagnosed in the mid- to late-stages of the disease when the rod cells are mostly lost. Therefore, the elucidation of the biological mechanisms that underlie retinal degeneration, especially in the secondary cone cell death phase, will be critical in developing novel treatments for RP, in addition to individualized gene therapy.

In RP, rods are expected to be injured because of gene mutations that are exclusively expressed or critically function in rod cells. However, why and how cones also die subsequent to rod cell death is puzzling. Accumulating evidence suggest that microenvironmental changes associated with rod cell death such as loss of trophic factors (117), metabolic alterations (118), oxidation (119), collapse of outer nuclear layer (120), and inflammation (121), which are associated with rod cell death, may contribute to the secondary cone cell death. These factors mutually influence each other. For example, cytokines or chemokines released from a collapsed retina evoke inflammatory cell activation and migration, activated microglia or macrophages produce reactive oxygen species (ROS) to enhance oxidative stress, and, alternatively, oxidative stress triggers, or augments inflammatory response. The roles of oxidative stress and metabolic dysfunction in cone cell death in RP have been summarized in a previous review (119, 120). The present review focuses on inflammation and immunobiology as potential factors that mediate and modulate cone degeneration in RP. Assessing immune involvement in RP/IRDs from a broader perspective, including RP cases in the rod degeneration phase, is beyond the scope of this review and have been described in our previous recent review (11).

### Clinical Findings of the Inflammatory Response and Its Relationship With Cone Degeneration in RP

Cell death and inflammation have a tight interaction with each other. Dying or dead cells stimulate phagocytes to mediate their clearance and maintain tissue homeostasis, whereas excessive activation of inflammatory cells can exert cytotoxicity and exacerbate the disease (122).

Without exception to this scenario, inflammatory cell infiltration is usually observed in the vitreous of RP patients. Using vitreous samples from post-mortem RP patients, Newsome et al. demonstrated that this cell infiltration consists of a mixed component of inflammatory cells including monocytes, NK cells, lymphocytes, and others cells (123). In a previous study, the authors of the present review graded the severity of inflammation in the anterior vitreous using *vivo* in slit-lamp biomicroscopy, and found that RP patients with a higher number of inflammatory cells had worse visual acuity and lower central retinal sensitivity (121). We also evaluated aqueous flare, a marker of blood-ocular barrier breakdown and inflammation. Our study data showed that aqueous flare values are increased in the eyes of RP patients, compared to these values in healthy individuals, and that aqueous flare values are negatively correlated with central visual function in RP patients (124). Consistent with our observation, independent groups have reported that aqueous flare values have a negative association with visual field area (125) and a positive correlation with inner retinal thickening, which occurs during retinal degeneration and remodeling, in RP patients (126). Daylight vision in the central and peripheral area is provided by cone cells; therefore, these findings suggest that inflammation may be implicated in retinal degeneration, especially in secondary cone cell death that occurs in RP. However, these studies were cross-sectional clinical studies and have the limitation that a cause-effect relationship could not be elucidated.

Cytokines and chemokines have critical roles in evoking the differentiation, activation, migration, and suppression of immune cells. A comprehensive measurement of inflammatory cytokines and chemokines in the aqueous humor and vitreous of the eyes of RP patients using multiplex enzyme-linked immunosorbent assay showed that IL-6, IL-8, and MCP-1 are elevated in the aqueous humor of RP patients, and that a greater variety of molecules (e.g., IL-1β, IL2, IL-4, IL-6, IL-8, IL-10, IFN-γ, MCP-1) are increased in the vitreous with more significant fold changes (121). Lu et al. also conducted multiple cytokine analyses and observed increased IL-6, IL-8, and MCP-1 levels and increased extracellular matrix-related proteins such as matrix metalloproteinases (127).

The inflammatory cytokines/chemokines elevated in RP are related to innate and acquired immunity. IL-1β, IL-8, and MCP-1 are pivotal molecules for activating and recruiting monocytes/macrophages and neutrophils to the inflammatory loci. IFN-γ, IL-2, IL-4, and IL-10 are produced primarily or partly by T lymphocytes, and they mediate the differentiation and polarization of Th cells and macrophages. These profiles of inflammatory cytokines/chemokines in RP are consistent with the infiltration of a variety of inflammatory cells into the vitreous, as described above (123). To develop an anti-inflammatory therapy for RP, further studies are needed to elucidate the key inflammatory cytokines/chemokines that critically contribute to the disease progression.

High sensitivity CRP (hs-CRP) is a serum inflammatory marker, and an increased hs-CRP is associated with age-related macular degeneration, DR, and uveitis (128, 129). The measurement of serum hs-CRP levels in RP patients without systemic disorders revealed that hs-CRP levels are ~2 times higher in RP patients than in control subjects (130). In addition, a higher hs-CRP level is associated with a faster deterioration in central retinal sensitivity in RP patients (130). Taken together, these findings suggest that peripheral immune cells and ocular resident immune cells may be implicated in the disease progression of RP.

### Functional Roles of Inflammatory Response in Cone Cell Death in RP

The findings outlined previously suggest that innate and acquired immunity are activated and involved in the pathology of RP. However, the function of each inflammatory cells (e.g., microglia, macrophages, and lymphocytes) and its regulatory mechanisms remains unclear and is a topic of interest.

Microglia, a resident macrophage in the CNS that derived from the embryonic yolk sac progenitors, are the most prominent immune cells in the retina (131). Microglia are long-lived cells that persist throughout the entire lifetime of mice, and can proliferate and repopulate after experimental depletion or during retinal degeneration (132). Monocytes in the peripheral blood do not invade the CNS in healthy conditions, although they can infiltrate and differentiate into macrophages in an aging or diseased retina with a dysfunctional blood-retinal barrier (133). These monocyte-derived macrophages resemble microglia in their morphology and their long life span, but have different functional features such as lower expression of colony-stimulating factor 1 receptor and higher expression of proinflammatory molecules such as MHC-II, IL-1β, and TNF-α (134). These two myeloid cells (i.e., tissue-resident microglia and monocyte-derived macrophages) have been extensively investigated as pivotal innate immune cells that contribute to the health and disease of the retina.

Several reports have demonstrated the detrimental function of microglia/macrophages in retinal degeneration in experimental RP. In a previous study, suppression of activated microglia/macrophages with minocycline or toxin-induced depletion of CX3CR1-positive microglia/macrophages protected rod cells against cell death in retinal degeneration (rd) 10 mice (135, 136). However, attenuation of the homeostatic function of microglia by disrupting the CX3C chemokine ligand 1-CX3C chemokine receptor 1 axis or the complement component 3–complement receptor 3 axis accelerates rod degeneration, along with proinflammatory microenvironmental changes such as increased TNF-α and IL-6 levels (137, 138). Therefore, microglia/macrophages have a bidirectional function in RP, as expected from the basic understanding of the interaction between cell death and inflammation. Dissection of the protective and detrimental populations among microglia/macrophages and precise understanding of the differential function of microglia and macrophages in RP warrant further studies. In addition, clinical studies suggest a link between inflammatory markers and cone function; therefore, the effect of microglia/macrophages on cone cell death also requires further study.

Oxidative stress significantly contributes to cone cell death in RP. Campochiaro et al. postulated that rod cell loss in RP substantially reduces oxygen consumption in the retina, and the remaining cone cells are exposed to a high-level of oxygen and resultant ROS (119). They showed that oxidized proteins, lipids, and nucleic acids are accumulated in the outer retina (139), and that pharmacological or genetic suppression of oxidative stress leads to significant rescue of cone cells in animal models of RP (140).

**Figure 3:**
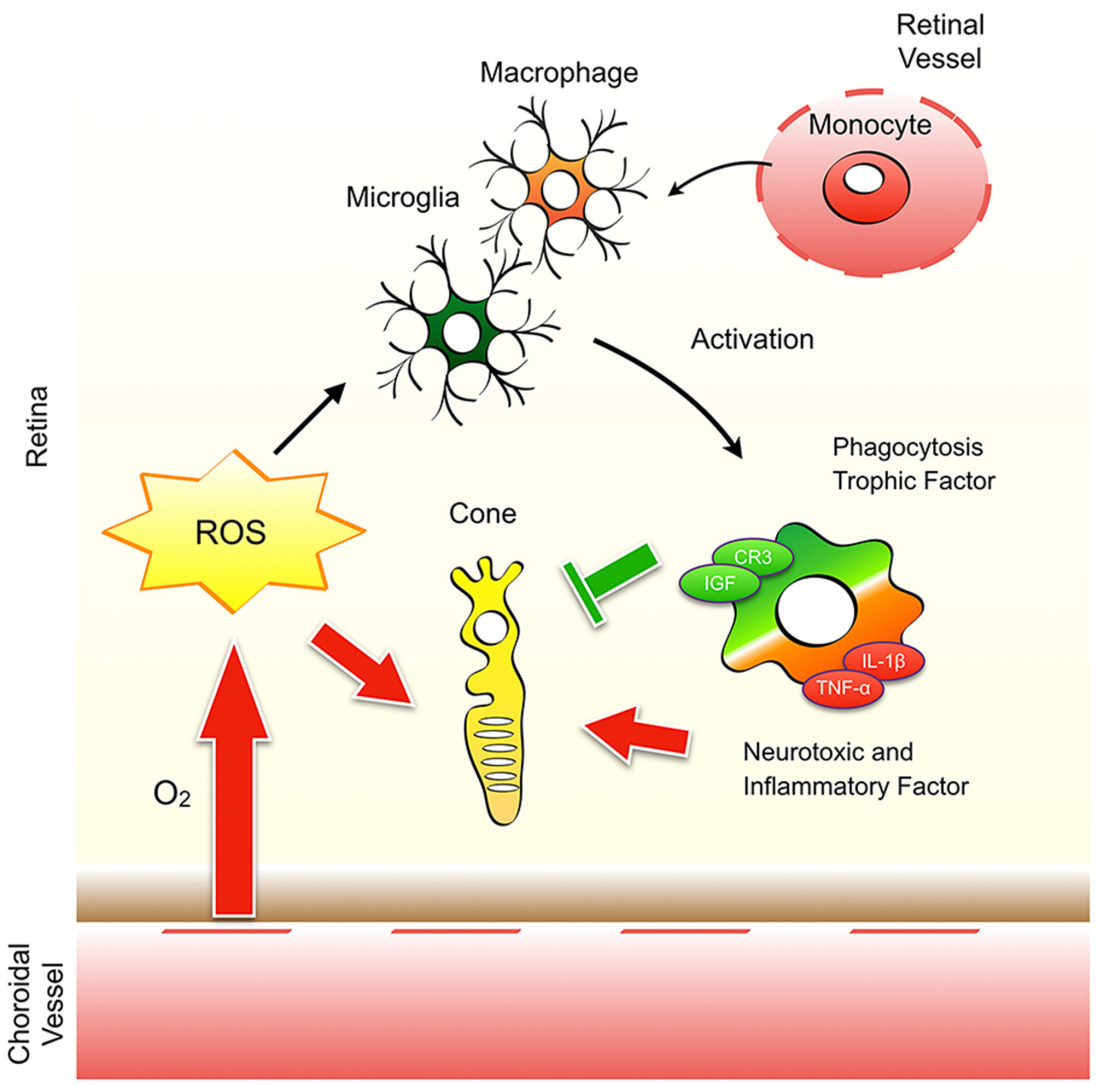
Oxidative stress modulates cone cell survival and neuroinflammation in retinitis pigmentosa. The remaining cones in retinitis pigmentosa (RP) are exposed to a high level of oxygen and the resultant reactive oxygen species (ROS). ROS have a direct harmful effect on cone cells and affects the activation of microglia and monocyte-derived macrophages. Activated microglia/macrophage have a bidirectional function to protect or promote cone cell death. Which environmental factors (e.g., ROS, molecules released from dead cells) and cellular factors (e.g., microglia vs. macrophage) are critical to determine the homeostatic vs. neurotoxic function of microglia/macrophage in RP is unclear.

Oxidative stress may have a direct harmful effect on cone cells, but it also affects microglial/macrophage activation in RP. In a previous study, we showed that treatment with anti-oxidant N-acetylcystein (NAC) substantially suppresses microglia/macrophage activation with reduced MCP-1, IL-1β, RANTES, and TNF-α expression (141). The anti-inflammatory effects of anti-oxidants are also observed in eyes with experimental retinal detachment that are treated with a free radical scavenger, edaravone (142). Our study further demonstrated that oxidative activation of microglia/macrophages is a key step in the augmentation of retinal inflammation and degeneration (including cone cell death) in rd10 mice. This activation is partly mediated by an oxidative DNA repair enzyme, MUTYH; an excessive activation of which leads to the formation of single strand breaks and increased expression of TNF-α in microglia/macrophages (143). The concept that oxidative stress alters homeostasis vs. neurotoxic balance of microglia and modulates cone cell survival and neuroinflammation in RP is shown in Figure 3.

Lymphocytes and lymphocyte-related cytokines are increased in the eyes of RP patients. In addition, several studies suggest the possible involvement of an autoimmune response and antiretinal autoantibodies in the progression of RP (144). However, the roles of acquired immunity in RP have been less investigated than the role of innate immunity.

Rohrer et al. crossed rd1 mice with *Scid* or *Rag1*^−/−^ mice, both of which lack functional T and B lymphocytes, and showed that the deficiency of lymphocytes did not change rod cell death (145). In another study, Mishra et al. demonstrated that rod degeneration was mildly attenuated in rd1 *Nod.Scid* mice, which are deficient in T, B, and NK cells (146). These findings suggest that NK cells may play a minor role in rod cell death in RP, whereas lymphocytes may not have a significant function, at least solely by themselves. However, lymphocytes and their related molecules/factors are clinically observed in the mid- to late stage of RP. Thus, studying the roles of acquired immunity in cone cell death will be important in future research.

## Vitreoretinal Lymphoma

Most primary vitreoretinal lymphomas (PVRL), which were previously termed as intraocular lymphomas, are related to high-grade non-Hodgkin's lymphoma, which is a subset of primary central nervous system lymphomas (PCNSL) (147). In Japan, 21 per 100,000 patients with ocular disorders have PVRL (148). PVRL comprises 1% of non-Hodgkin's lymphoma and <1% of intraocular tumors (149). Most cases of VRL are primary or secondary to CNS disease or may present simultaneously with it; however, it can also rarely be derived from systemic metastatic lymphoma (150). PVRL usually occurs in adults from the fifth to the sixth decades of life (151). No sex or racial predilection to the disease apparently exists, although some reports proposed that PVRL occurs more frequently in females than males (152).

### Clinical Features of Vitreoretinal Lymphoma

The clinical ocular features of VRL, termed “masquerade syndrome,” are often similar to those of chronic uveitis; therefore, a misdiagnosis of VRL sometimes leads to the administration of anti-inflammatory agents such as corticosteroids and thereby cause a delay in reaching a definitive diagnosis. The interval between the onset of the ocular or neurological findings and a definitive diagnosis is variable, and ranges from 4 to 40 months (152). The involvement of the CNS arises in 16–34% of patients with PVRL at presentation and develops in 42–92% of patients within a mean interval 8–29 months (151). Ocular involvement occurs in 15–20% of patients with PCNSL (151). Most patients with VRL have bilateral ocular involvement, but they often present with unilateral involvement at the initial visit owing to the uneven distribution of the disease. PVRL usually develops in the retina, the vitreous chamber, and/or the optic nerve, but can sometimes involve the anterior segment of the eye (153). Vitritis is the most common sign in VRL, and the findings has an “aurora borealis”-like appearance. In VRL, responses to corticosteroid therapy is initially observed but treatment resistance subsequently occurs. Multifocal whitish to yellow subretinal infiltrates are often observed. Coalescence of the lesions with “leopard skin”-like pigmentation, which is characteristic of VRL, is sometimes observed.

The overall survival of VRL patients has improved over the decades because of earlier diagnosis of the disease, which is a result of advances in molecular biological or genetic techniques. In addition, intense systemic chemotherapy and/or radiotherapy have also increased the overall survival rate. Several prospective studies have recently demonstrated that high-dose methotrexate (HD-MTX)-based chemotherapy with intravitreral MTX injections, subsequent whole brain radiotherapy (WBRT), and/or consolidation chemotherapy could improve overall survival and prevent CNS progression (5–7). Kaburaki et al. showed that a combination treatment protocol of intravitreral MTX injections, MTX-based systemic induction chemotherapy and consolidation high-dose cytarabine, and subsequent reduced-dose WBRT for the treatment of PVRL accomplished a 4-year progression-free survival of 72.7% and a 4-year overall survival of 88.9% (7), which suggests that these intensive systemic therapeutic modalities should be introduced for CNS prophylaxis. Some cases are resistant to these regimens, although no standard regimens exist for the treatment of refractory and relapsed PCNSL. Retreatment with HD-MTX-based chemotherapy has been administered in some patients. WBRT or high-dose chemotherapy and autologous stem cell transplantation in younger patients who have not undergone these treatments as part of the first-line therapy are used as a salvage therapy instead (154). However, introducing these treatments to elderly patients and patients with a poor general condition is often difficult to prevent CNS progression and to treat PCNSL because of the possible occurrence of treatment-induced adverse events such as neurotoxicity and nephrotoxicity (155, 156). In the next section, we review the association of pathogenesis, especially the genetic or immunological aspects, with B-cell VRL to exhibit new diagnostic and/or therapeutic targets for the treatment of VRL.

### Gene Mutations in Vitreoretinal Lymphoma

Most cases of PVRL can be classified as diffuse large B-cell lymphoma (DLBCL), whereas very few cases are classified as a T cell lymphoma or NK cell origin PVRL (152). Based on gene expression profiles, DLBCL is divided into three major subgroups: germinal center B-cell-like, activated B-cell-like (ABC)/non-germinal center, and primary mediastinal DLBCLs (157). The immunophenotype of most PCNSLs resembles ABC-DLBCLs, which are more aggressive and have poor prognostic outcomes, compared to the others (158).

High frequency of myeloid differentiation primary response gene 88 (MyD88) and CD79B mutations have been characterized in PCNSL (159). A single leucine-to-proline substitution at amino acid position 265 of Myd88 (*MYD88* L265P), is the most common mutation and accounts for more than 60% of VRLs (160). MYD88 is the adapter protein that mediates intracellular signaling pathways downstream of the toll-like receptor and the IL-1 receptor families. The CD79B mutation occurs in 35% of patients with PVRL, and is associated with the CNS progression of PVRL (161). The B-cell receptor (BCR) complex-associated protein β chain, CD79B, forms a complex with BCR and generates after recognition of an antigen to activate chronic BCR signaling. *MYD88* L265P and/or CD79B mutation contribute to the constitutive activation of NF-kB or BCR signaling, thereby promoting tumor growth.

Furthermore, in cases of PCNSL, because of the HLA locus mutation (chromosome 6q21.32), a shortage in HLA class I and II expression on tumor cells leads to escape from T or NK cell-mediated immune surveillance against tumor cells (162), which suggests that the lack of immune recognition of foreign antigens is one of the mechanisms that B-cell VRL cells preferentially retains in the eye.

PD-1, which is expressed on activated T-cells such as cytotoxic T lymphocytes (CTLs), interacts with its ligands (PD-L1 and PD-L2). These ligands are commonly expressed on tumor cells and upregulated in the tumor microenvironment (TME), thereby promoting inhibitory signaling of T cell receptors (TCRs) in CTL and subsequent tumor growth (163, 164). In PCNSL, investigations of copy number variations have revealed that frequent copy number gains at chromosome 9q24.1, which contains the PD-L1/PD-L2 locus (159). Chromosomal translocation involving the PD-L1/PD-L2 locus were also discovered in PCNSL, which indicates that immune evasion may be associated with the development of PCNSLs, including PVRL.

### Diagnosis of Vitreoretinal Lymphoma

Cytological examination of the intraocular fluid or tissue is the gold standard for a definitive diagnosis of VRL. However, cytology alone can have a low diagnostic yield (40–60%) because of the limited amount of specimen that can be obtained, necrosis, and the fragility of VRL cells (165, 166). The vitrectomy cell block technique can improve diagnostic yield and can be utilized for immunohistochemistry of pan B-cell markers, including CD20 and CD79a, to establish a definitive diagnosis of lymphoma (167–169).

Several supplementary diagnostic methods can improve the definitive diagnosis of VRL. They include cytokine analysis to determine the ratio of IL-10 to IL-6 (i.e., the IL-10/ IL-6 ratio) (170), molecular analysis of the immunoglobulin heavy (IgH)/ TCR chain gene to confirm monoclonality, and flow cytometric identification of cell surface markers (171, 172). However, clonal expansions of lymphocytes have not been circumscribed in VRL. Therefore, molecular analysis with polymerase chain reaction and flow cytometric identification can sometimes yield false-positive results (173, 174).

Furthermore, *MYD88* L265P can be screened with new genetic techniques, including allele-specific polymerase chain reaction and next generation sequencing (NGS) using an oncogene gene panels, which allows for lower cellularity or a smaller volume of samples to confirm the definitive diagnosis of VRL (175, 176).

### Etiopathogenesis in Vitreoretinal Lymphoma

As an exogenous factor, infection with the Epstein-Barr virus (EBV) is associated with PCNSL, specifically in immunocompromised patients such as individuals with acquired immune deficiency syndrome (AIDS) (177). EBV, which is a ubiquitous human herpes virus, affects most of the human population. EBV infects humans mostly in childhood and early adulthood, and subsequently spreads to B-lymphocytes and exists in a latent state. In patients with impaired cell-mediated immunity, such as patients with immune suppression and the elderly with immunosenescence, latent EBV may proliferate indiscriminately and drive neoplastic transformation to lymphoid malignancy (178). EBV, *Toxoplasma gondii*, and human herpes virus 8 are speculated as a cause of PVRL due to the detection of their gene expression in the intraocular fluids of some patients with PVRL (179).

On account that PVRL has selective tropism to CNS lesions, a theory has been proposed that chemokines and their receptors encourage the attraction and maintenance of VRL cells in intraocular tissues. In patients with B-cell chemokines, CXCL12, and CXCL13 are specifically expressed in retinal pigment epithelial cells and/or in the vitreous cavity of patients with VRL. As a consequence, B-lymphoma cells [which express CXCR4 and CXCR5 (i.e., receptors for CXCL12 and CXCL13)] are recruited into intraocular tissues such as the retina, vitreous body, and subretina (180). CXCL13 levels are also increased in the vitreous humor of patients with VRL (181). CCL19 derived from astrocytes was recently reported to promote the retention of lymphoma cells, which express CCR7, and subsequent tumor growth in chronic gliosis lesions in mice (182).

### Immune Evasion in Vitreoretinal Lymphoma

VRL cells can evade attacks by CTLs and NK cells because the eye is an immune-privileged site that possesses an immunosuppressive ocular microenvironment composed of soluble and cell surface inhibitory molecules. TGF-β is abundant in the vitreous humor to maintain an anti-inflammatory state in the eye (8). IL-10, which is widely regarded and has been analyzed as an immunosuppressive cytokine, has a pivotal role in the induction of immune tolerance in the eye (183). In cancer, regulatory cytokines in the TME such as IL-10 and TGF-β support tumor development by suppressing antitumor immunity in cancer (184, 185). Treg cells, which are a highly suppressive subset of T cells, increase regulatory cytokines secretion for the maintenance of self-tolerance and inhibition of autoimmunity, which result in tumor development (186, 187). In B-cell malignancies, including systemic DLBCL, the TME, which is formed with the reactive T-cells, macrophages, stromal cells, blood vessels, and extracellular matrix, regulates tumor cell survival or proliferation, and immune evasion for treatment resistance, associated with worse prognosis (188). Vitreous samples of B-cell VRL contain a large number of benign T cells and macrophages as well as tumor cells (174), which suggests that the infiltrating immune cells form the TME and support the suppression of anti-tumor immunity in the vitreous body.

### Advances in the Treatment for Vitreoretinal Lymphoma

Based on the outcome of the genetic studies described previously, several new agents are currently being investigated as a salvage therapy in clinical trials assessing in patients with refractory or relapsed PCNSL and PVRL. In a prospective French multicenter phase II trial, monotherapy with ibrutinib, which targets Bruton's tyrosine kinase downstream of BCR, was effective and had objective response rates (ORRs) of up to 70% (189). Among 14 PVRL patients, the median progression-free survival (PFS) was 22.7 months. The median overall survival was not estimated because more than one-half of the PVRL patients were alive (Table 2). Immunomodulatory agent monotherapy with lenalidomide, which inhibits the NF-kB and PI3K/AKT pathways, achieved ORRs of up to 64% (192). However, in a prospective clinical study (191), lenalidomide in combination with intravenous rituximab for refractory or relapsed PCNSL and PVRL maintained an ORR of 35.6% owing to the short response to the therapy. Among 11 PVRL patients, the median PFS was 9.2 months. Nivolumab, an anti-PD-1 agent, has been reported to have responses and maintain complete remission in patients with relapsed/refractory PCNSL (193). In the future, the combination of these agents with MTX-based chemotherapy should be assessed as the first-line therapy for PVRL.

**Table 2:** Salvage treatment regimen for PVRL in prospective clinical trials.

**Agent**	**Number of patients**	**Median PFS (mo.)**	**Median OS (mo.)**
CYVE + ASCT (190)	5	8	19.2
Ibrutinib (189)	14	22.7	Not estimated
Lenalidomide + rituximab (191)	11	9.2	Not reported

*PVRL, primary vitreoretinal lymphoma; PFS, progression free survival; OS, overall survival; CYVE, high-dose cytarabine and etoposide; ASCT, autologous stem cell transplantation*.

Furthermore, increased expression of proinflammatory cytokines related to CTLs, such as IFN-γ, granzyme A, and IP-10 occurs in the aqueous humor and/or vitreous of VRL patients (181), which suggests that CTLs are associated with the pathogenesis of VRL. In a previous study, we revealed that subretinal infiltration of VRL cells elicits the infiltration of T-cells into the vitreous cavity (194). However, we were unable to elucidate the association of the T cells with the prognosis of the VRL patients. Little data exist that elucidate the roles of reactive T cells in DLBCL-VRL. In the future, further detailed studies on the infiltration of T cells into the eyes of VRL patients may provide new insights into the pathogenesis of the disease and deliver new therapeutic targets such as augmentation of CTL and/or NK cells function.

## Conclusion and Future Directions

A profound understanding of the intricacies of immune responses will raise innovations for the management and treatment of these intractable retinal disorders. The generation of biologics, including IFX or ADA, has dramatically changed the treatment of NIU in the past few decades. However, during long-term treatment of NIU patients, a decreased response or adverse events to the biologics has emerged because of the development of antidrug antibodies or paradoxical effects. The development of selective small-molecule therapies is expected to resolve these problems. From the results of our analysis of EAU, the induction of regulatory DCs may be useful for the treatment of NIU.

In retinal vascular diseases, low-grade inflammation can destroy vascular integrity by the action of VEGF and some cytokines/chemokines from infiltrated leukocytes. Resistance to anti-VEGF therapy is sometimes observed in DR (including DME); therefore, developing a new therapy associated with low-grade inflammation as a “beyond VEGF” therapy for retinal vascular diseases, including DR and ROP, may be useful.

Immunological responses also affect the pathogenesis of RP, despite differences in genetic backgrounds. Targeting cytokines/chemokines associated with immunological responses against RP may be an attractive target for the treatment of RP, in addition to gene therapy.

The recent introduction of molecular profiling technologies, including NGS, can exhibit the molecular characterization of several cancers to provide information on tumor diagnosis and specific targeted therapy. Several agents, which were selected on the basis of the molecular characterization, have been assessed in clinical trials of cases of refractory/relapsed PCNSL; however, the utility of these molecular profiling technologies has not been established. Considering the rarity of VRL, large-scale collaborative registries, tumor molecular profiling programs, and clinical trials in institutions across the world are necessary to enhance diagnosis, prognostication, and treatment outcomes in the future.

## Author Contributions

All authors listed have made a substantial, direct and intellectual contribution to the work, and approved it for publication.

## Conflict of Interest

The authors declare that the research was conducted in the absence of any commercial or financial relationships that could be construed as a potential conflict of interest.
